# Tertiary care for infertile couples: aetiological diagnoses and conventional treatment outcomes in Kisangani, Democratic Republic of the Congo

**DOI:** 10.1186/s40834-023-00236-w

**Published:** 2023-07-18

**Authors:** Jean-Didier Bosenge-Nguma, Antoine Modia O’yandjo, Juakali Sihalikyolo, Noël Labama Otuli, Kadima Ntokamunda, Batina Agasa, Katenga Bosunga

**Affiliations:** 1 Department of Obstetrics and Gynecology, Faculty of Medicine and Pharmacy, University of Kisangani, P.O. Box 2012, Kisangani, Democratic Republic of the Congo; 2 Department of Clinical Pharmacology, University of Rwanda, Kigali, Rwanda; 3 Department of Internal Medicine, Faculty of Medicine and Pharmacy, University of Kisangani, Kisangani, Democratic Republic of the Congo

**Keywords:** Infertility, Tubal factors, Anovulation, Clomiphene citrate, Conventional treatment, Kisangani

## Abstract

**Background:**

In sub-Saharan Africa, tubal factors are described as the main aetiological factors of infertility. Under these conditions, medically assisted procreation is particularly indicated. However, Assisted Reproductive Technology centres are less available. Thus, infertile couples are quickly oriented towards available alternative conventional treatments. The present study aimed to determine the aetiological factors of infertility, the outcomes of the therapeutic options offered, and the factors associated with the success of conventional treatment among infertile couples seeking tertiary care in Kisangani.

**Methods:**

A cross-sectional study was conducted at two tertiary health facilities in Kisangani. Infertile couples who provided consent underwent specific examinations necessary for the exploration of infertility and were treated and followed up for a minimum of 6 months. The therapeutic options that were offered were expectant attitude, medical treatment, surgical treatment or transfer to an in vitro fertilization unit. The pregnancy diagnosis was performed by ultrasound.

**Results:**

A total of 272 infertile couples underwent specific examinations, were treated and were followed up for a minimum of 6 months. Many determinant causes were mostly linked to wives rather than husbands. Overall, only 34 women among 211 who were treated became pregnant during the follow-up period; 61 couples were advised to resort to IVF or adoption, but the couples for whom expectant the attitude was indicated immediately rejected it. The patients who therapeutically succeeded at the end of the treatment were those who were younger than 35 years (OR = 2.27; 95% CI = 1.06–4.87; *P* = 0.017), had a duration of infertility of less than five years (OR = 6.08; 95% CI = 1.79–20.69; *P* = 0.001) and had secondary infertility (OR = 6.08; 95% CI = 1.79–20.69; *P* = 0.001).

**Conclusion:**

Kisangani faces a major issue in the treatment of infertility. Treatment of patients using conventional methods is limited by the predominance of tubal factors as aetiological determinants of infertility. The low pregnancy rate found in this study provided additional evidence of this. This paper represents a serious plea to national policy-makers to encourage them to pay attention to issues surrounding infertility.

## Introduction

Sociologically speaking, the deep longing of any married couple is to consolidate their commitment by the arrival of children as quickly as possible. If children do not arrive soon, anxiety takes over, and the excitement fades away. An infertile couple is thus characterized by the woman's inability to become pregnant, which leads to several psychological and social consequences, such as fear, guilt, depression, anxiety, self-blame, marital stress, emotional abuse, intimate partner violence, divorce, abandonment by the partner, social isolation, economic deprivation, and loss of social status, in some regions (e.g., Africa and Asia) and can even cause starvation, exposure diseases, violence-induced suicide and loss of dignity in death [1–3].

Medically, infertility is generally defined as the inability to conceive after at least 12 months of regular and proper sexual intercourse without contraceptive methods [4, 5]. Because the fertility rate among women is known to decline steadily with age, it is advised to evaluate and treat women aged 35 years or older after six months of unprotected sex [5].

There are two types of infertility, primary and secondary infertility. Primary infertility refers to couples who have not been able to conceive after at least one year of having regular sex without using birth control methods, while secondary infertility refers to couples who have been able to become pregnant at least once, regardless of the outcome (abortion, premature, full-term or post-term delivery) or the site of implantation of the pregnancy (ectopic or intrauterine pregnancy), but who are no longer successful.

As noted by the World Health Organization (WHO), infertility is a global health issue that affects millions of people of reproductive age worldwide [6]. However, the incidence of cases varies considerably, mainly depending on each people’s cultural reproductive health behaviours and the definition used in different studies (clinical-1 year, epidemiological-2 years, demographic-5 years) [6].

Available data suggest that 804 million women aged 20–44 years (122 million in developed countries and 682 million in underdeveloped countries) are married or in consensual unions. Among them, 72.4 million have clinical infertility, of which 40 million are likely to seek health care, and 32.6 million will not seek health care for the management of infertility [7]. According to WHO data, more than 180 million couples in developing countries suffer from primary or secondary infertility [8]. A systematic analysis of 277 health surveys [6] showed that in 2010, 1.9% of women aged 20–44 presented with primary infertility and 10.5% with secondary infertility. The researchers found that the levels of infertility were similar in 1990 and 2010, with only a slight overall decrease in primary infertility (0.1%, but with a more pronounced drop in SSA and South Asia) and a modest overall increase in secondary infertility (0.4%). In SSA, the prevalence of secondary infertility declined from 13.5% in 1990 to 11.6% in 2010.

Common causes of infertility include lack of regular ovulation, poor-quality semen, blocked or damaged fallopian tubes, and endometriosis. The WHO's International Classification of Diseases provides more information on the many primary and secondary causes of infertility in women and men [5]. Infertility may occur due to male factors, female factors, or a combination of male and female factors or may be unexplained. However, for both women and men, environmental and lifestyle factors such as smoking, excessive alcohol intake, obesity, and exposure to environmental pollutants have been associated with lower fertility rates [9].

Although prevention of infertility and education about reproductive health are top priorities, there is a great demand for novel assisted reproductive technologies (ARTs) and accessible diagnostic techniques [4]. The availability, cost, and efficacy of ARTs must be maximized in resource-poor contexts such as Africa, notwithstanding the fact that only approximately 1.5% of the population is said to have access to them.

There is no evidence from studies in low- or middle-income countries that low-cost ARTs are effective, accessible, and affordable to most people in need of these services. In addition, in sub-Saharan Africa, tubal factors are described as the main aetiological factors for infertility as a result of sexually transmitted infections (STIs), unsafe abortions and complications of childbirth [10]. Under these conditions, medically assisted procreation is particularly indicated [11].

However, few programs specialize in infertility management in SSA, including the Democratic Republic of Congo (DRC). Reproductive health programs have generally focused on contraceptive methods [12]. Infertility and ARTs are not considered priorities in many low- and middle-income countries (LMICs), particularly in SSA. The most often used arguments against the use of ART are overpopulation, other health priorities (e.g., family planning, vaccinations, malaria, etc.), limited government budgets and limited experience of providers with inadequate facilities for performing sophisticated procedures [13]. In some LMICs, ART is considered expensive and only moderately effective, with risks of complications and unknown effects on women and their offspring [13]. In addition, religions, cultures and health care systems constitute other obstacles to the use and advancement of ART [14]. Thus, infertile couples are quickly oriented towards available alternative conventional treatments [15].

The present study aimed to highlight the aetiological factors, outcomes of available therapeutic options, and factors associated with the success of conventional treatment among infertile couples treated in tertiary care facilities in Kisangani in the northern DRC.

## Methods

### Study type and setting

This was a cross-sectional study conducted from July 1, 2019, to December 31, 2022, at two tertiary health facilities, namely, the University Clinics of Kisangani and ‘’Clinique les Anges Kisangani’’, public and privately owned facilities, respectively. These two structures offer specialized care in several areas, particularly in fertility, and receive the majority of infertile couples from Tshopo and neighbouring provinces.

### Ethical issues

The two hospitals follow a standard protocol for investigating and managing all infertile patients. Before conducting this study, we obtained the approval of the research ethics committee of the University of Kisangani (UNIKIS/CE/018/2019) and the authorizations of the managers. Participation was voluntary and anonymous and based on written consent. Refusal to participate in this study did not impact the infertile couple's access to appropriate care.

### Study population size

During the study period, 798 couples consulted with a desire to conceive. Of these, 297 completed all the examinations required for the standard aetiological workup of infertility, including 272 who agreed to participate in this study. We excluded couples who refused to participate in this study, those in whom one or both partners were unable to complete all of the examinations required in this study to explore the aetiological factors of infertility (a frequent situation in our context), and those who voluntarily dropped out. Infertility was defined as the absence of conception after at least 12 months of regular and complete sexual intercourse without contraceptive methods [2]. The investigation procedure and treatment options for infertility were the same for all patients, both those who agreed to participate in this study and those who did not.

### Diagnostic explorations

The protocol included three essential components: medical history, physical examination, and paraclinical explorations. Table 1 summarizes the data collected from the patients' clinical histories.

**Table 1:** Data collected from the patients' clinical histories

Female	Male
Demographics (age, profession, education…) Gynaecological history (pubertal development, menstrual history, coital age, frequency and time of sexual intercourse, sexual disorders, change of partner, sexually transmitted infections (STIs), etc.) Obstetric history (parity, gestation, abortion, uterine curettage, endo-uterine infection, galactorrhoea, etc.) Medical history (history of chronic diseases, long-term treatment or hormonal treatment, history of pelvic surgery, etc.)	Demographics (age, profession, education …) Developmental history (puberty development, cryptorchidism and testicular torsion, inguinal or testicular surgery) Infection history (orchitis, orchid epididymitis, mumps, STIs …) Medical history (chronic diseases, previous treatments, etc.)

Physical examinations were performed, with emphasis on the examination of the genital apparatus. The specific paraclinical examinations carried out for women were transvaginal ultrasound, hormonal assay and hysterosalpingography (HSG). HSG was scheduled and performed according to the protocol described in the study we conducted in 2019 [16]. For men, the genital examination included the location, number and size of the testicles, as well as the presence of varicocele or any other congenital or acquired genital abnormality. The paraclinical examinations systematically requested were spermogram and infectious assessments. Semen analysis was performed after 3–5 days of sexual abstinence, and the results were interpreted according to the WHO laboratory manual [17].

### Conventional treatment of infertile couples

After finishing the investigations, an appointment was set with the couple to discuss the results obtained and the treatment to be administered, taking into account the available resources. Depending on the results of the investigations, the therapeutic options were expectant attitude, medical treatment, surgical treatment or transfer to an in vitro fertilization (IVF) unit. However, when discussing treatment, most couples (~ 91%) for whom expectant attitude was indicated immediately rejected this option for multiple reasons to be explored in the future; hence, this treatment option was ruled out.

After initiation of treatment, the couple was followed up for at least six months before evaluating the results in terms of conception (clinical pregnancy). The diagnosis of clinical pregnancy consisted of ultrasound visualization of at least one gestational sac with foetal heartbeat.

### Data analysis

Data were analysed using IBM SPSS version 22 software (IBM Corp., Armonk, NY, USA). Qualitative data are described as proportions, quantitative data are described as the means with their standard deviations (SDs). Pearson's chi-square test was used to compare ratios with a significance level of *P* < 0.05.

## Results

### Participant sociodemographic characteristics and histories

A total of 272 infertile couples were included in this study. The history characteristics of the patients are presented in Table 2. The women's mean age (± SD) was 32.85 years (± 6.02) and that of the men was 43.47 years (± 9.65). Most women (58.09%) had a secondary education, while the husbands (61.4%) had a high school education. Eighty-eight (32.35%) couples had primary infertility, and 184 (67.65%) had secondary infertility. The average duration of infertility (± SD) at the time of the couple's hospital visit was 6.09 years (± 4.80). Most women were nulliparous (63.97%) and had a monogamous union (66.91%). Only 26.47% had a history of abortion, and 59.56% had a history of pelvic surgery.

**Table 2:** Sociodemographic and clinical characteristics of patients

		**Females**		**Males**	
Variables	Strata	N	%	N	%
Age	< 35 years	166	61.03	40	14.70
	≥ 35 years	106	38.97	232	85.30
	Mean (± SD)	32.85(± 6.02)		43.47(± 9.65)	
Educational level	Primary	6	2.21	4	1.47
	Secondary	158	58.09	101	37.13
	High school	108	39.70	167	61.40
Type of marriage	Monogamous	182	66.91		
	Polygamous	90	33.09		
Parity	Nulliparous	174	63.97		
	1–2	80	29.41		
	3–4	18	6.62		
History of abortion	Yes	72	26.47		
	No	200	73.53		
History of pelvic surgery	Yes	162	59.56		
	No	110	40.44		
Type of infertility	Primary	88	32.35		
	Secondary	184	67.65		
Duration of infertility	≤ 5	162	59.56		
Mean ± SD (6.09 ± 4.80)	> 5	110	40.44		

### Identified aetiological factors of infertility

**Fig. 1:**
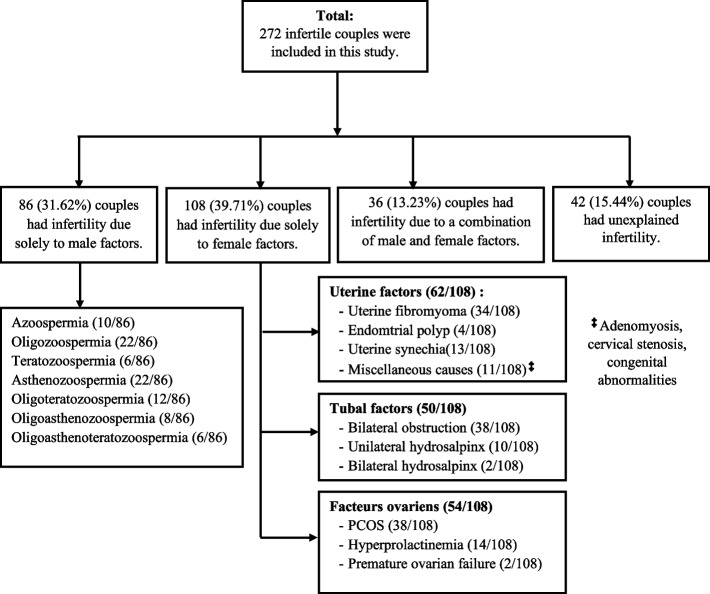
Flow diagram describing the aetiological factors of infertility in Kisangani

The aetiological factors identified after investigations of infertile couples are detailed in Fig. 1. Of all the couples, 31.62% (86/272) had infertility due solely to male factors, 39.71% (108/272) had infertility due to female factors, and 13.23% (36/272) had infertility due to a combination of male and female factors. In 15.44% of couples, the cause of infertility remained unexplained. In women, the predominant causes were tubal factors (50/108), anovulation caused by polycystic ovarian syndrome (PCOS) (38/108) and hyperprolactinemia (14/108), and uterine leiomyoma (34/108). The most commonly identified tubal factors were bilateral tubal obstruction (38/108), unilateral hydrosalpinx (10/108), and bilateral hydrosalpinx (2/108).

Male factors were represented by abnormalities in semen analysis (86/272). Indeed, among the 86 men with abnormal semen analysis, 10 (11.63%) had azoospermia, 22 (25.58%) had oligozoospermia, 6 (6.98%) had teratozoospermia, 22 (25.58%) had asthenozoospermia, 12 (13.95%) had oligoteratozoospermia, 8 (9.3%) had oligoasthenozoospermia, and 6 (6.98%) had oligoasthenoteratozoospermia. One hundred six members of infertile couples also had evidence of seminal infection. A link between the type of infertility and the category of aetiological factors was also found (Table 3). Indeed, male factors were significantly associated with cases of primary infertility (*P* = 0.0004), while female factors were mainly associated with secondary infertility (*P* = 0.0018).

**Table 3:** Relationship between causes and types of infertility

Causes of Infertility	Primary infertility		Secondary infertility		*P* value
	N	%	N	%	
Female factors	24	27.27	84	45.65	0.0018
Male factors	40	45.45	46	25.00	0.0004
Combination of factors	20	22.73	16	8.70	0.0011
Unexplained causes	4	4.55	38	20.65	0.0001
Total	88	100.0	184	100.0	

### Conventional treatment options offered to couples and outcomes

Conventional treatment options included pharmacological treatment and surgical procedures. Details of the treatment options offered to infertile couples in the two facilities that served as the study setting are presented in Table 4. For women, pharmacological treatment mostly consisted of ovulation induction with oral ovulogens, including clomiphene citrate as a partial oestrogen agonist alone (29.86%) or in combination with bromocriptine (antihyperprolactinemia), metformin (insulin sensitizer-like), or letrozole (aromatase inhibitor). Men received mainly hormonal treatment (2%), antioxidants (18%) and antibiotic therapy according to the results of the bacteriological culture (50.4%). Surgical treatment consisted of myomectomy/polypectomy (11.37%) and tubal surgery (7.58%). In addition, tubal flushing was performed in 14 patients (6.64%).

**Table 4:** Therapeutic options offered to 211 couples after infertility assessment

Therapeutic options administered	N	%
Medical treatment offered to women		
Ovulation induction with clomiphene	63	29.86
Ovulation induction with letrozole	23	10.90
Ovulation induction with clomiphene + metformin	8	3.79
Ovulation induction with clomiphene + bromocriptine	4	1.90
Ovulation induction with clomiphene + metformin + bromocriptine	2	0.95
Treatment with bromocriptine alone	10	4.74
Procedures/surgery performed on women (Given alone or in combination with medication)		
Myomectomy/Polypectomy	24	11.37
Tubal flushing	14	6.64
Tubal surgery (salpingostomy/fimbriolysis/adhesiolysis)	16	7.58
Therapeutic options available to men		
Hormonal treatment	4	1.90
Antibiotics (genital infection)	106	50.24
Antioxidants	38	18.00
Total couples treated	211	100.0

There were 36 (17.06%) positive urine pregnancy tests from the 211 couples who were treated and followed up. A total of 34/211 (16.11%) individuals had their pregnancy sonographically confirmed. Table 5 provides a list of the variables that influenced the outcome of infertility treatment. Patients who were younger than 35 years (OR = 2.27; 95% CI = 1.06–4.87; *P* = 0.017), had infertility lasting less than five years (OR = 6.08; 95% CI = 1.79–20.69; *P* = 0.001), and those who had secondary infertility (OR = 6.08; 95% CI = 1.79–20.69; *P* = 0.001) were significantly more likely to become pregnant at the end of treatment. Additionally, compared to couples whose infertility was caused by male factors or a mix of male and female factors, couples whose infertility was caused by a female factor or those whose infertility was caused by an unexplained reason had a higher chance of becoming pregnant at the end of treatment. A monogamous or polygamous marriage had no effect on how well the infertile couples responded to treatment.

**Table 5:** Factors associated with the success of conventional infertility treatment (*n* = 211 cases)

Factors	Strata	Outcome (pregnancy)				OR	[95% CI]	*P* value
		Success		Failure				
		N	%	N	%			
Patient’s age	< 35 years	25	21.37	92	78.63	2.56	1.13–5.80	0.010
	≥ 35 years	9	9.57	85	90.43			
Infertility type	Primary	3	4.35	66	95.65			
	Secondary	31	21.68	112	78.32	6.08	1.79–20.69	0.001
Marriage type	Monogamous	21	14.79	121	85.21	0.74	0.34–1.59	0.228
	Polygamous	13	18.84	56	81.16			
Infertility duration	< 5 years	22	21.78	79	78.22	2.27	1.06–4.87	0.017
	≥ 5 years	12	10.90	98	89.09			
Infertility causes	Female	21	25.00	63	75.00	4.77	1.82–12.52	0.000
	Male + Female	6	6.52	86	93.48			
	Unexplained	7	20.00	28	80.00	3.58	1.11–11.55	0.019
All factors	Total	34	100.0	177	100.0			

## Discussion

### Aetiological diagnosis of infertility

A total of 272 infertile couples underwent specific examinations necessary for exploring infertility and were then treated and followed up for a minimum of 6 months. Infertility was defined as the absence of conception after at least a year of regular, proper sexual activity without the use of contraception. Only 34 women became pregnant during the monitoring period. The majority of determinant factors were related to women rather than to men. Similar studies have been conducted worldwide, and our results show comparable results and specific particularities.

In this study, 67% of the couples presented with secondary infertility and 33% with primary infertility, consistent with the results from SSA countries [12, 14, 18–20]. The proportions of patients with primary and secondary infertility vary according to the aetiological particularities of each geographical region. A plausible explanation for the predominance of secondary infertility in SSA is the high prevalence of tubal lesions [12, 20]. Several studies have reported a significant association between tubal pathology and secondary infertility [21, 22]. We previously reported the presence of tubal lesions in 72.31% of infertile patients in Kisangani in 2019 [16]. Another study conducted in Goma [18] found 67.6% of tubal factors among infertile patients. In SSA, tubal factors are described as the main aetiological factor of infertility [11]. The main reason for this high proportion of patients with tubal blockage is more likely due to the high prevalence of pelvic inflammatory diseases among women in these environments secondary to STIs, unsafe abortions and complications of childbirth [10].

Notwithstanding this high prevalence of tubal factors, several other African studies [14, 23, 24] have widely reported the high prevalence of ovulatory disorders. For example, Adewunmi et al. [14] reported 43.5% of ovarian factors versus 33.7% of tubal factors in Ikeja, Nigeria. In Sudan, Elhussein et al. [24] noted anovulation in 52.34% (178/342) of cases of female infertility.

Female causes have generally been the most studied in Africa because women are believed to be the primary cause of infertility [25]. However, several studies have shown that male factors contribute to infertility in a nonnegligible proportion [26, 27]. Malekshah et al. [27] reported male factors in 38.9% of infertile couples, and approximately half of the husbands had STIs. Additionally, approximately 26% of women had a history of abortion. Our study further noted that male factors were significantly associated with cases of primary infertility (*P* = 0.0004), while female factors were predominantly associated with secondary infertility (*P* = 0.0018). The same observation was made by Ekwere et al. in Calabar [28].

The patients’’mean age (± SD) was 32.85 ± 6.02 years. This mean age is similar to those in two other Congolese (DRC) studies conducted by Kadima et al. [19] and Mboloko et al. [15] in Mbuji-Mayi (32 years) and in Kinshasa (33.7 ± 5.2 years), respectively. The same mean age was also reported in Sudan (32.4 ± 7.4 years) [24]. Other authors have found a slightly higher median age in Nigeria (38.9 ± 5.2 years) [14] and India (36.7 years) [29]. In SSA, most girls get married between 18 and 25 years of age because it is widely accepted that fertility in women declines steadily with age. The age limit for pregnancy acceptable for women is generally 35 years for young couples. This study showed that the chance of treatment success was 2.5 times higher for women under the age of 35 than for those older than 35.

Furthermore, the mean infertility duration found in our study was 6.09 ± 4.80 years, greater than the demographic (5 years), epidemiological (2 years) and clinical (1 year) definitions of infertility [6]. Mittal et al. [29] reported an average infertility duration of 9.1 years in India. The average age of infertile patients and the average duration of infertility described in the present study attest that most infertile couples consult the gynaecologist late when the chance of spontaneous natural birth has decreased [30]. In the DRC, most infertile couples prefer to consult general practitioners and traditional practitioners first. The gynaecologist is often consulted only when traditional treatment fails or rarely on the recommendation of a general practitioner [15].

Another factor that compromised pregnancy in this study was a history of pelvic surgery in 59% of women. Previous studies conducted in the DRC reported a history of pelvic surgery in 65.6%, 49.59% and 40.1% of infertile patients in Kinshasa [12], Goma [18] and Mbuji-Mayi [19], respectively. One of the reasons for the high proportion of infertile patients with a history of abdominopelvic surgery in the DRC seems to be the route they follow to find infertility care. Indeed, Mboloko et al. [15] found in a study conducted in an urban setting that in the DRC, infertile patients consult first-line health workers who are not qualified in the management of infertility. This behaviour most often exposes them to incorrect and dangerous practices, such as unjustified abdominopelvic surgery with the aim of exploring the ovaries and fallopian tubes.

### Treatment outcomes

Among the factors that caused female infertility, the present study indicated ovulatory disorders such as PCOS (38/108 patients) and hyperprolactinemia (14/108 patients). Therefore, pharmacological treatment was the primary treatment option for both men and women, and the medications used were those commonly recommended in therapeutic practice to normalize ovulation. The patients who therapeutically succeeded at the end of treatment were those who were younger than 35 years, those with a duration of infertility less than five years and those with secondary infertility.

Another medication suggested in the literature that we did not use is inositol supplementation [31, 32]. Myo-inositol could be a valid insulin-sensitizing molecule for managing menopausal disorders. It is the precursor to inositol triphosphate, acting as an intracellular second messenger and regulating several hormones, such as thyroid-stimulating hormone (TSH), follicle-stimulating hormone (FSH) and insulin [31]. Espinola et al. [32] presented 2 cases of successful ovulation with d-chiro-inositol in non-PCOS, noninsulin-resistant young women, likely by modulating aromatase expression.

At the end of the investigations, 61/272 (22.43%) couples were referred to an in vitro fertilization (IVF) unit or, failing that, advised to adopt. These were couples in which one partner, or both, had one or more severe infertility factors such as premature ovarian failure, severe tubal or uterine factors (bilateral tubal obstruction, hydrosalpinx, extensive synechia, etc.), major congenital malformations of the genital system or severe anomalies in the spermogram. Additionally, considering the expensive cost of the procedure, only six couples attended IVF centres and completed the course. Among them, two conceived. The therapeutic outcomes of these couples followed up in IVF centres were not considered in this study's results.

Additionally, we did not seek the causes of the refusal of the adoption of children. However, in most African societies, the success of marriages is largely conditional upon procreation, and women are often victims of childlessness. Adoption, which could be an alternative strategy for infertile couples, is not very widespread [33]. Studies indicate that barriers to adopting children in Africa include cultural practices, negative reactions from husbands, psychological dissatisfaction, family dynamics, financial implications, lack of information about adoption and procedural problems [33–35]. A study by Ti-enkawol et al. [35] reported that the adoption of a child was a sign of acceptance of defeat in the quest for biological children. The same authors found that family dynamics that may impede child adoption include the high value of blood ties, the unpredictable influence of the adopted child's family, discrimination against the adopted child and the fact that the family does not allow the adopted child to inherit their property. Raising awareness, mobilizing the community and enacting supportive legislation that protects all parties involved would help stem negative attitudes against the practice of adoption.

Assisted reproduction remains inaccessible in many parts of the world, particularly in sub-Saharan Africa, where most countries still do not have IVF clinics [36]. In the DRC, for example, almost all patients who undergo IVF have to pay out of pocket for medical treatment and IVF procedures since there are no public subsidies for ARTs in this country. To our knowledge, the median price of an IVF cycle in the DRC, including drugs, is 8000 US dollars. ARTs are, therefore, not affordable for the average Congolese couple. In addition, geographically, the distribution of IVF centres does not facilitate access to all patients. The few IVF centres in the DRC are private and located in Kinshasa, the country's capital city. In addition to having the means to pay for treatment, most patients must be able to take time off from work and/or family obligations and travel long distances for treatment. Sociocultural and religious barriers are also obstacles to the acceptance and rapid implementation of ARTs in Africa [37]. Taking all of the above into account, the study by Afferri et al. [38] suggests recognition of infertility as a disease, solid and proactive political commitment, and reduction of care costs through public‒private partnership opportunities as the three pathways to facilitate the inclusion of fertility care in African reproductive health policies. However, given the economic realities in sub-Saharan Africa, the guidance provided by the study by Inhorm et al. [36] seems concrete and promising. First, these authors propose preventing infertility through early detection and treatment of reproductive tract infections, including STIs such as gonorrhoea and chlamydia, as well as postpartum and postabortion infections and iatrogenic infections. Infertility prevention also involves health education on metabolic diseases (overweight/obesity, insulin resistance/diabetes and polycystic ovary syndrome). Second, the authors propose ways to destigmatize infertility and support infertile patients who find themselves ostracized in societies where parenthood is socially obligatory, such as in sub-Saharan Africa.

In this way, cultural and religious myths and hurdles can also be overcome. Finally, the authors advocate for the adoption of low-cost IVF initiatives (LCIVF) in low-resource nations. The goal of LCIVF is to make in vitro fertilization (IVF) safe, affordable, efficient, and accessible to infertile couples living in resource-constrained nations. For this, Ombelet et al. [39, 40] created the simplified low-cost IVF culture system. Similar to traditional intracytoplasmic sperm injection (ICSI), this cutting-edge low-cost IVF method produces outcomes.

Additionally, this innovative technique offers the possibility of bringing IVF to a large portion of the world's population, making it accessible to the great majority of infertile patients, thanks to its low cost. It thus symbolizes a glimmer of hope for the growth and expansion of IVF in sub-Saharan Africa. Injecting embryo culture supernatant into the endometrial cavity had no effect on the outcomes of IVF/ICSI or oocyte donation cycles, according to a randomized clinical experiment [41]. Despite the lack of a definite clinical advantage, costs could increase.

Measurements for this experiment did not include knowledge, attitudes, or psychological factors. Some researchers claim that patients frequently do not fully understand the genetic causes of their sickness, and thus infertility is considered a stimulant for psychological morbidity [42].

### Limitations

The results of this study may not be generalizable to the whole population of the DRC because it was conducted in an urban population. The fact that patient follow-up after therapy began lasted only six months was another limitation of this study. If the follow-up was prolonged, the success rate of the treatment may also have increased. A pregnancy incidence of 40.1% was observed in a Nigerian study [43] that followed up patients for ten years.

One advantage of this study is that all of the patients who were included in it underwent the tests necessary for the conventional aetiological assessment of infertility. Another is that the data collection was prospective.

## Conclusion

This study is pertinent because the WHO maintains that infertility in Africa needs to be studied [44]. The management of infertility is a considerable challenge in Kisangani. The preponderance of tubal factors as aetiological factors does not allow great success with conventional treatment. The low pregnancy rate found in this research served as confirmation of this. However, increasing public knowledge of the risks associated with tubal lesions and the importance of seeking timely medical attention at specialized facilities can help improve treatment outcomes. This report constitutes a serious request to motivate national policy-makers to take an interest in infertility concerns.

## Data Availability

The datasets used and/or analysed during the current study are available from the corresponding author on reasonable request.

## Abbreviations

ART: Assisted Reproductive Technology
ARTs: Assisted Reproductive Technologies
HSG: Hysterosalpingography
IVF: In Vitro Fertilization
ICSI: Intra Cytoplasmic Sperm Injection
<: Lower than
≥: Greater than or equal to
CI: Confident Interval
DRC: Democratic Republic of the Congo
LCIVF: Low-Cost In Vitro fertilization
OR: Odd Ratio
PCOS: Poly Cystic Ovarian Syndrome
SD: Standard Deviation
SSA: Sub-Saharan Africa
STIs: Sexually Transmitted Infections
WHO: World Health Organization
